# India fights hard to neutralize the spread of COVID-19

**DOI:** 10.1017/ice.2020.140

**Published:** 2020-04-16

**Authors:** Govindasamy Agoramoorthy

**Affiliations:** College of Pharmacy and Health Care, Tajen University, Yanpu, Pingtung, Taiwan

*To the Editor—*A novel coronavirus (SARS-CoV-2) that originated in Wuhan, China, has created a pandemic across 198 countries over the first few months of 2020.^1^ As of April 5, 2020, India has 3,072 confirmed cases, 213 recovered persons, and 75 deaths, and more new cases are emerging rapidly. India has a huge population of >1.3 billion people, and cities such as Delhi, Mumbai, Kolkata, Chennai, Bangalore, Hyderabad, and Pune harbor millions of people who rely on public transportation. The government has aggressively promoted social distancing to minimize the spread of this virus.

On a daily basis, millions of people pass through crowded train stations such as Delhi, Howrah, Sealdeha, Mumbai, and Chennai. For example, the Sealdha station alone receives 1.8 million passengers, and most are from low- and middle-income families that depend on intracity transportation. Such close contact among people in highly crowded areas is potentially catastrophic for community spread of the virus. In response to this crisis, the government has created expert groups to tackle the practical problems on the ground. For example, both international and domestic flights have been grounded.^2^

Few detection centers to screen for SARS-CoV-2 currently exist, so a transportation chain is necessary to take samples (eg, sputum, blood, urine, and nasal swabs) from collection points to testing centers. Several days are required to obtain test results. In addition, false-positive and false-negative results can occur and must be carefully avoided. The country’s elite Indian Council of Medical Research should create more detection and observation centers to facilitate a more rapid testing process. Through agencies such the National Institute of Virology in Pune, the government has tried to bring factual awareness regarding the virus and to eliminate the spread of false information via social media. However, this effort needs support from all healthcare NGOs to encourage people to remain calm and to act rationally.

India’s pharmaceutical industries are also facing difficulties because they obtain 70% of all active pharmaceutical ingredients from neighboring China, where the pandemic originally started.^3^ In addition, pharmaceutical trading companies depend on finished products from China such as nebulizers, high-filtration masks, and thermometers. Due to the shortage of products, goods are either unavailable in drug stores or are sold at high prices, beyond the budget of low- and middle-income households. Similarly, hand sanitizer is scarce in drug stores. Even though the government is educating people to use masks and hand sanitizer, the shortage of materials is a concern that must be dealt with swiftly.

The Indian government has implemented a strict and timely quarantine policy for returning workers, either in a hospital or at home. Violators are prosecuted by law, and adhering to strict discipline has become a crucial mandate. Furthermore, spraying alcohol on roads, vehicles, public trains, and personnel to disinfectant people has no value. Vast quantities of alcohol spray are detrimental to human health.^4^ Health education must provide advice based on scientific evidence. The spread of unscientific information (eg, drinking cow urine to counter the coronavirus) must be totally stopped. Close monitoring to facilitate a better understanding of the epidemiology and transmission pattern of the SARS-CoV-2 virus across all states is vital. The government needs to consider the effectiveness of public health policies in terms of their social implications in practice.^5^

Both central and state governments across India have taken several scientific control measures to weed out the spread of the SARS-CoV-2 virus. The prime minster also initiated a disaster fund for the South Asian Association for Regional Cooperation (SAARC) block nations to assist neighboring nations. Within India, several states have allocated a special fund to deal with the pandemic. On March 25, 2020, a total lockdown of all states across the nation was undertaken for 21 days to control the community spread of the virus. The World Health Organization has praised India’s response. India should continue the massive efforts against the SARS-CoV-2 virus along with the already successful “Clean India” campaign promoted by the prime minister because adopting better hygiene may contribute to minimizing the spread of this dangerous pandemic.
